# Treatment of Breast and Prostate Cancer by Hypofractionated Radiotherapy: Potential Risks and Benefits

**DOI:** 10.1016/j.clon.2015.02.008

**Published:** 2015-07

**Authors:** K.J. Ray, N.R. Sibson, A.E. Kiltie

**Affiliations:** Cancer Research UK and Medical Research Council Oxford Institute for Radiation Oncology, Department of Oncology, University of Oxford, Oxford, UK

**Keywords:** α/βratio, breast cancer, clinical trials, hypofractionation, prostate cancer, radiotherapy

## Abstract

Breast cancer and prostate cancer are the most common cancers diagnosed in women and men, respectively, in the UK, and radiotherapy is used extensively in the treatment of both. *In vitro* data suggest that tumours in the breast and prostate have unique properties that make a hypofractionated radiotherapy treatment schedule advantageous in terms of therapeutic index. Many clinical trials of hypofractionated radiotherapy treatment schedules have been completed to establish the extent to which hypofractionation can improve patient outcome. Here we present a concise description of hypofractionation, the mathematical description of converting between conventional and hypofractionated schedules, and the motivation for using hypofractionation in the treatment of breast and prostate cancer. Furthermore, we summarise the results of important recent hypofractionation trials and highlight the limitations of a hypofractionated treatment regimen.

## Statement of Search Strategies Used and Sources of Information

The review is based on a search of peer-reviewed publications found using Web of Science, PubMed and Google Scholar. The reference lists from some articles were also used to obtain other pertinent articles. Information on relevant clinical trials was obtained from the International Clinical Trials Registry Platform.

## Introduction

Breast and prostate cancer are the most common cancers, accounting for 30 and 25% of cancer diagnoses in women and men, respectively, in the UK. Efforts to improve survival in these two cancers have been highly successful; breast cancer 5 year survival in England has improved from 52% in 1971–1975 to 85% in 2005–2009, whereas the 5 year survival in prostate cancer has improved from 31 to 81.4% over the same time periods. This improved outcome is largely due to increasing awareness of these diseases, the National Health Service Breast Cancer Screening Programme and stage migration in the diagnosis of prostate cancers [1]. At the same time, radiotherapy has emerged as an important treatment modality for both breast and prostate cancer. This review discusses the risks and potential benefits of using hypofractionated radiotherapy schedules, which have been hypothesised to improve treatment outcome owing to the specific properties of each of these two cancers.

## Radiotherapy Treatment, Normal Tissue Effects and the Linear Quadratic Model

Radical radiotherapy treatment regimens involve dividing the overall radiation dose into a number of fractions, as this takes advantage of the five Rs of radiotherapy: reoxygenation of hypoxic cells, repopulation of cells, repair of normal tissues, redistribution of cells through the cell cycle and differences in the intrinsic radiosensitivity of tumour cells compared with normal tissue cells [2]. A typical conventional fractionation regimen uses 2 Gy fractions delivered daily five times a week, up to a total of 60–80 Gy, although this varies across treatment centres, tumour types and patients [3].

**Fig 1:**
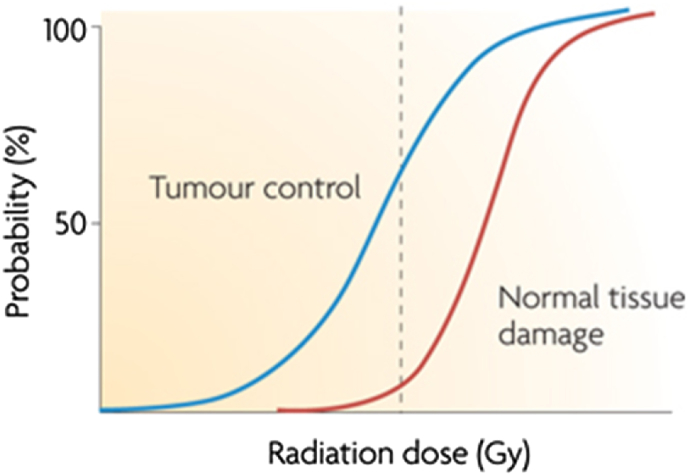
Sigmoid-shaped curves for tumour control (TCP, left) and normal tissue complication/damage (NTCP, right) probability. The dashed lines indicate a 60% TCP and a 5% NTCP for a given dose. Reprinted from [4] with permission from Macmillan Publishers Ltd.

A good therapeutic outcome in radiotherapy comprises both local control of the tumour and minimal late normal tissue complications. These can be quantified using the tumour control probability (TCP) and normal tissue complication probability (NTCP) curves [4] (Figure 1). The separation between the curves gives the size of the therapeutic index, which can be altered by shifting either the TCP or the NTCP curve. As the dose per fraction increases, the probability of toxic effects in late-responding normal tissues increases disproportionately compared with early responding normal tissues, which are typically less sensitive to fractionation [4]. The cause of the rise in late-responding normal tissue toxicity is not yet well understood, but may be due to a number of factors, including cell death and the production of cytokines [5]. Late-responding normal tissue toxicity can be further subcategorised into primary effects due to apoptosis of slowly proliferating cells and consequential effects due to initially acute damage of early responding normal tissue. It is primary late-responding normal tissue toxicity that typically limits radiotherapy treatment regimens [4].

Fractionation sensitivity can be quantified using a parameter known as the α/β ratio from the linear quadratic model, which is formally defined as the dose at which the proportion of cell killing due to lethal single-hit injuries (αD) and the accumulation of sublethal injuries (βD^2^) are equal. A small α/β ratio indicates tissue with a large sensitivity to changes in dose per fraction. This low α/β ratio can result from a number of intrinsic properties of the tissue, such as a greater capacity to repair DNA damage induced by radiation and a slow proliferation rate [6]. Hence, typically, late-responding normal tissues have a smaller α/β ratio (about 2 Gy) than either tumour tissue or early responding normal tissues (about 10 Gy) [7].

## Hypofractionated Radiotherapy

A hypofractionated schedule is one that delivers a dose larger than 2 Gy per fraction (with a lower overall dose). To convert between conventional and hypofractionated schedules, the biologically effective dose (BED) calculation, defined in equation (1) [8], is used. In this equation, D is the total dose prescribed and d is the dose per fraction. This allows clinicians to determine a dose equivalent to the standard prescribed dose for a new schedule, resulting in the same biological effect either on the tumour or the dose-limiting normal tissues.

(1) BED=D[1+dαβ]

For example, if a clinician prescribes a total of 60 Gy to be delivered to a tumour with an α/β ratio of 4 Gy (typical for breast tumour) in conventional 2 Gy fractions, the BED for this regimen is 60(1 + 2/4) = 90 Gy. To deliver the same BED of 90 Gy in 3 Gy fractions, however, we need only deliver fractions up to a total dose of 90/(1 + 3/4) = 51 Gy, which in 3 Gy fractions is only 17 fractions.

An extension to the BED is the equivalent dose in 2 Gy fractions, EQD_2Gy_, which is defined by equation (2) [9]. The EQD_2Gy_ of a 51 Gy in 3 Gy fractions hypofractionated regimen is 51(3 + 4)/(2 + 4) = 59.5Gy ≈ 60 Gy, as expected from the BED example above.

(2) EQD2Gy=D[d+αβ][2+αβ]

## Hypofractionated Radiotherapy in Breast and Prostate Cancer

Although hyperfractionation, the delivery of multiple doses smaller than 2 Gy to further spare late normal tissue effects, is of proven effectiveness in oropharyngeal cancers [10], hypofractionated schedules are potentially attractive in the treatment of breast and prostate cancer. The α/β ratios of these tumours are thought to be the same or less than the surrounding late-reacting normal tissues. Prostate tumours are estimated to have an α/β ratio of about 1–3 Gy [11] and breast tumours an α/β ratio of about 4 Gy [12]; this compares with oropharyngeal cancers and other head and neck squamous cell carcinomas, with α/β ratios of over 8 Gy [11]. These low α/β ratios are thought to reflect the characteristically slower proliferation rates of breast and prostate tumours compared with other tumour types. As a consequence of this slower proliferation, these tumours respond in a similar manner to late-responding normal tissue rather than early responding normal tissue [13–15], thereby removing the rationale for hyperfractionated schedules. Radiotherapy is usually used in breast cancer in the adjuvant setting, delivering whole breast irradiation by external beam radiotherapy after breast-conserving surgery to a lower dose than radical doses given at other tumour sites. The purpose of treatment is to eliminate any cancer cells not removed during surgery. In contrast, irradiation of the prostate is given to a higher radical dose either by external beam radiotherapy or brachytherapy [6,12].

## Benefits of Hypofractionation in Prostate and Breast Cancer

The primary benefit of treating breast and prostate cancer with a hypofractionated regimen is the improved therapeutic index that can be obtained. Hypofractionation can achieve this in one of two ways when compared with the conventional fractionated scheme [6]: (i) dose escalation to increase tumour control while maintaining the same normal tissue complication probability (i.e. shift the TCP curve to the left in Figure 1) or (ii) maintaining dose equivalence in terms of tumour cure probability while decreasing the normal tissue dose (i.e. shifting the NTCP curve to the right in Figure 1). Physical methods, such as intensity-modulated radiotherapy, three-dimensional conformal radiotherapy and charged particle therapy, have been used in conventionally fractionated radiotherapy to decrease the normal tissue dose [6] and their use minimises the toxicity of hypofractionated radiotherapy.

As well as the therapeutic gain of hypofractionation, a number of other advantages are conferred in terms of logistical, patient convenience and resource allocation considerations [16]. Reduced numbers of fractions will reduce radiotherapy costs in terms of work-hours and fewer fractions also results in fewer visits for a patient to the radiotherapy clinic, which is more convenient and less costly [17]. However, these advantages should be considered as ‘added bonuses’ to a hypofractionated schedule: its primary purpose should be for therapeutic gain. To this end, numerous clinical trials in breast and prostate cancer have been conducted. Although the aim of many of these trials was to show a therapeutic gain from using a hypofractionated schedule, others sought only to show that normal tissue toxicity is comparable for the conventional and hypofractionated treatment regimens. Therefore, as long as a hypofractionated schedule is no more toxic than the conventional schedule, it can be argued that the logistical advantages are sufficient to warrant clinical implementation.

Hypofractionation in breast cancer radiotherapy entered clinical trials in the UK in 1986 with the Standardisation of Breast Radiotherapy pilot trial (START-pilot; *n* = 1410) and in Canada in 1993 with the Ontario trial (*n* = 1234) [18]. These were followed in 1999 with the START-A and START-B trials [19]. These trials challenged conventional wisdom, which justified the rationale for 50 Gy in 25 fractions, namely that cancers in the breast are less sensitive to changes in dose per fraction than the dose-limiting surrounding normal tissues. They then tested the hypothesis that breast tumour tissue and surrounding late-reacting normal tissue are similarly sensitive to fraction size. The then-conventional 50 Gy in 25 fractions over 5 weeks was compared with 41.6 Gy or 39 Gy in 13 fractions over 5 weeks in START-A (*n* = 2236) and with 40 Gy in 15 fractions over 3 weeks in START-B (*n* = 2215), the latter being a dose-fractionation already commonly used in the UK. Primary end points were locoregional relapse and late normal tissue effects. The START-pilot and START-A trial were designed to allow estimation of the α/β ratios of breast tumour and late-responding normal tissue, and similar α/β ratios were found for breast tumour using locoregional relapse as the end point and surrounding normal tissue in the range 3.5–4.7 Gy [19] (Table 1). Ten year follow-up data are now available [19] and these found that breast shrinkage, telangiectasia and breast oedema were significantly less frequent with 40 Gy than 50 Gy in START-B, with no evidence that 40 Gy in 15 fractions was less efficient in achieving locoregional control. These trials resulted in the standard breast cancer radiotherapy treatment protocol in the UK changing to 40 Gy in 15 fractions [19].

The randomised UK FAST trial (2004–2007, *n* = 915) went further with its hypofractionation regimen, testing 50 Gy in 25 fractions over 5 weeks against either 30 or 28.5 Gy in five fractions over 5 weeks [20]. Acute tissue toxicity effects as well as late tissue effects were measured, with the primary end point being adverse effects in the breast. Where the START trials showed that the principle of hypofractionation in breast cancer treatment was effective, the FAST trial investigated how much the dose per fraction could be increased before adverse acute normal tissue reactions became intolerably high. After a 3 year median follow-up, the 28.5 Gy fractionation scheme was comparable with the 50 Gy scheme in terms of adverse reactions, and lower than the 30 Gy scheme because the total dose to the tissue was lower [20]. This trial has been followed by the FAST Forward trial, which closed 2 years earlier than expected after very rapidly recruiting 4000 patients. FAST Forward is testing 40 Gy in 15 fractions over 3 weeks against two accelerated hypofractionation schemes of 26 or 27 Gy in five fractions over 5 days. The decreased overall treatment time may improve tumour cure probability by decreasing the effect of tumour repopulation over the treatment time, but may also increase early responding normal tissue complications, although this could be mitigated by the decreased overall dose. The five-fractions-in-5-days schedule is hugely convenient to patients, and frees up large amounts of machine capacity in an era where, with increasing patient numbers, this is at a premium. Further hypofractionation may be possible, where a single fraction delivered to part of the breast is sufficient for tumour control [21]. The clinical trials referred to in this review are summarised in Table 2.

The breadth and success of clinical trials of hypofractionation in breast cancer is encouraging; for prostate cancer the situation is less clear-cut. A recent review of 16 prospective randomised clinical trials comprising a total of 1828 patients concluded that hypofractionation for prostate cancer should still only be used in a clinical trial setting, owing to the uncertainties associated with the measurement of the α/β ratio for prostate tumour tissue [3]. Nevertheless, studies have continued, with one phase III randomised trial in Australia (*n* = 217) reporting a long-term therapeutic advantage in terms of biochemical relapse-free survival and also genitourinary side-effects at 4 years. Here the authors compared hypofractionated radiotherapy, using 55 Gy in 20 fractions over 4 weeks, to conventional radiotherapy, comprising 64 Gy in 32 fractions over 6.5 weeks, at a median follow-up of 7.5 years [22]. A recent meta-analysis of seven trials of 5969 patients, from a radiobiological perspective, gave an estimate of the α/β ratio for biochemical relapse-free survival of 1.4 (95% confidence interval 0.9–2.2) Gy [23], supporting the strategy of hypofractionation in prostate cancer.

The Conventional versus Hypofractionated High-dose intensity-modulated radiotherapy for Prostate cancer (CHHiP) study (*n* = 3216) between 2002 and 2006 in the UK compared the standard 74 Gy in 37 fractions over 7.5 weeks against two hypofractionated schedules: 60 or 57 Gy in 20 or 19 fractions, respectively [24]. Although still in active follow-up, preliminary analysis of 457 patients using grade 2 toxicity in the bowel or bladder on the Radiation Therapy Oncology Group (RTOG) scale as an end point seems to suggest that the hypofractionated schedules are safe and cause no more early/late normal tissue complications than the conventional regime 2 years after treatment [24]. The study design is also such that an estimate of the α/β ratio should be possible, and with 3216 patients in the trial it is thought the result will have a narrower 95% confidence interval than previous estimates. Further clinical trials either ongoing or in follow-up in Canada (‘PROFIT’, ISRCTN 43853433), the Netherlands (‘HYPRO’, ISRCTN 85138529), Scandinavia (‘HYPO-RT-PC’, ISRCTN 45905321) and the UK (‘PACE’, NCT01584258) will also contribute to a further enhancement in the precision of α/β estimates. These trials are summarised in Table 3.

## Risks Associated with Hypofractionation in Breast and Prostate Cancer

To some extent the hypofractionation approach is less toxic to normal tissue in breast cancer than in prostate cancer, as for the former lower relative doses are used in the adjuvant rather than radical setting. The improved therapeutic ratio when using a hypofractionated schedule is based on the assumption that the α/β ratio in breast and prostate cancer is lower than that of the surrounding normal tissue. Although measurements of α/β have been carried out using clinical data, these represent population averages and suffer from very large 95% confidence intervals reflecting large inter-patient variability [11,19]. The START-A breast cancer hypofractionation trial recruited 2236 women, but still yielded notably large 95% confidence intervals (Table 1) [19]. One prostate hypofractionation trial [11], which used a combination of external beam radiotherapy with high dose rate brachytherapy delivered in two or three implants, measured an estimated α/β of 1.2. However, because only 192 patients were recruited to their trial, the 95% confidence interval on this value was 0.03–4.1 Gy. The inter-patient variability of α/β values suggests that not all patients will benefit from the same hypofractionated schedule.

Assuming that breast tumour α/β ratio estimates are correct, there may be a limit to the extent of hypofractionation that can be used safely. For example, patients in the RAPID trial that underwent hypofractionated treatment with multiple fractions per day (38.5 Gy in 10 fractions over a week) suffered from greater normal tissue toxicity than those in the control arm (42.5 Gy in 16 fractions) [25]. The increased toxicity could be a result of many factors, including insufficient time for normal tissue to repair between fractions and the prescribed dose being delivered to too large a volume [25]. The FAST Forward trial may help to elucidate the lower limit of hypofractionation for breast cancer treatments.

Early prostate hypofractionation trials yielded low α/β ratios on comparing tumour control rates obtained with external beam radiotherapy and low dose rate brachytherapy [26,27]. However, these trials are limited in their power by the fact that they compare two therapy modalities as opposed to two different schedules of the same therapy modality, as well as the poorer dose conformity available at the time [6,27]. In fact, the findings of some recent trials using modern radiotherapy techniques are actually rejecting the hypothesis that hypofractionation is beneficial [3]. The lack of sufficient follow-up time has hindered many modern prostate cancer hypofractionation trials as they cannot adequately report on late-responding normal tissue complications, freedom from biochemical failure or morbidity. One multicentre international study, in 2003–2007, reported equivalent late toxicity effects between the hypofractionated and conventional treatment arms and significantly higher freedom from biochemical failure for the hypofractionated arm (88% versus 76%, *P* = 0.014), but only after a 3 year follow-up [28]. Such equivalence also limits the precision of any estimations of the α/β ratio, which in turn may also be dependent on the stage of the prostate tumour [6].

If the α/β ratios are not accurately estimated and doses selected appropriately, a hypofractionated schedule, as with all radiotherapy treatments, may increase normal tissue complications unnecessarily and potentially induce secondary cancers. One study comparing the late-responding normal tissue complications in prostate cancer radiotherapy found that, for patients with initial urinary problems, there was an increased frequency of complications following a hypofractionated radiotherapy schedule when compared with a conventional schedule [29]. An assumption is also being made that the linear quadratic model is valid at the sizes of dose per fraction used in clinical hypofractionated treatments. Although not a major issue in trials involving doses per fraction up to 3 Gy, the FAST Forward trial for breast cancer hypofractionation used a dose per fraction of over 5 Gy. The validity of the linear quadratic model at a high dose per fraction is yet to be confirmed [6,30–32].

Another factor that has not been considered in many clinical studies is the heterogeneous nature of tumour tissue [33]. Most tumours have both poorly and well-differentiated cells [34]; this heterogeneity leads to a heterogeneous α/β ratio across the tumour site (poorly differentiated cells have a higher α/β than well-differentiated cells). Consequently, a hypofractionated treatment could be sparing poorly differentiated sections of the tumour relative to the nearby organs at risk. Simulation of this effect showed that treatment efficacy decreases for a hypofractionated schedule even if 5–10% of the cells in the tumour are poorly differentiated [33]. The variation in genetic mutations across the tumour volume also contributes to its heterogeneity; it has previously been shown that there is as much inter-patient variability as there is intra-patient variability when considering the genetic mutations in biopsies of many tumour types [35]. These genetic mutations affect the ability of DNA repair pathways to function after radiation damage, hence affecting the α/β ratio.

Tumour heterogeneity also has implications on the use of biochemical relapse-free rate as an end point for measuring successful treatment of prostate cancer, as poorly differentiated cells produce less prostate-specific antigen than well-differentiated tumour cells [36]. Prostate cancer-specific survival or local tumour control and a long follow-up time period (10–20 years) are more robust end points. Heterogeneity of cell oxygenation within a tumour is also an issue; hypofractionation can result in reduced cell killing in hypoxic regions of tumour tissue [37]. Arguably, the importance of heterogeneity of the α/β ratio in tumour tissue is a factor that has been underestimated when determining the efficacy of hypofractionation treatment schedules.

## Discussion

Hypofractionated therapy schemes have obvious logistical and patient convenience advantages for individuals with breast or prostate cancer. However, although the breast cancer trial results are encouraging, the therapeutic gain in prostate cancer is less clear. This difference is mainly due to the measurement uncertainty of the α/β ratio for prostate tumour tissue. Although *in vitro* work with cell lines has supported a lower tumour α/β than the surrounding normal tissue for both cases [38,39], these results have not been conclusively replicated in human studies of prostate cancer. This variability may reflect insufficient follow-up time or patient numbers to report a precise result, suboptimal choice of end points or the radiotherapy modality used.

Hypofractionation in breast cancer treatment is now the standard protocol in the UK. The results of further trials investigating the limits of hypofractionation, such as the five-fractions-over-5-days regimen of the FAST Forward study, are eagerly awaited. Judging by the success of breast cancer hypofractionation, if we could get to a point where the α/β ratio for prostate cancer can be measured *in vivo* in a reliable manner and shown to be lower than surrounding organs at risk, hypofractionated therapy will probably have a beneficial impact for prostate cancer patients.

In order to address the issue of heterogeneity of tumour tissue, intensity-modulated radiotherapy could be used to increase the dose delivered to poorly differentiated areas of the tumour, but this would require *in vivo* imaging of cell differentiation. Adaptive radiotherapy, where the dose distribution is altered during the treatment schedule to account for changes in patient anatomy, tumour volume or areas of tumour that respond later in the course of treatment, may be used to provide more personalised and, ultimately, more efficacious treatment for each individual patient [40]. Alternatively, novel agents might be developed to alter the protein expression of selected parts of the tumour, thereby rendering it homogeneous in terms of radiotherapy response. If areas of tumour with a high α/β could be targeted based on genetic mutations that impair DNA repair capability, and this capability manipulated to decrease the α/β ratio, the tumour may respond more effectively to hypofractionated schedules. Moreover, calculations of therapeutic gain when using a hypofractionated schedule should be carried out using a model that accounts for the presence of hypoxia in tumours so as not to overestimate the cell killing, as can occur when using the linear quadratic model.

## Future Directions

Future steps in the clinical implementation of hypofractionated schedules will involve the development of biomarkers to measure individual α/β ratios for each patient and personalise treatment schedules to provide the optimum outcome. Estimates of α/β are currently based on population studies and variation between patients may have a significant effect on treatment efficacy for individuals. An ideal situation would be to measure the α/β ratio in each patient, while factoring in tumour heterogeneity, to enable prediction of the response to a particular treatment regimen, ultimately personalising the radiotherapy schedule to maximise the therapeutic index for each patient. This is a complex, and active, area of research.

## Figures and Tables

**Table 1:** α/β ratios estimated from START-A trial [19]

End point	α/β ratio (Gy)	95% confidence interval
Locoregional relapse∗	4.0	0.0–8.9
Locoregional relapse†	3.5	1.2–5.7
Breast shrinkage	3.5	0.7–6.4
Breast induration	4	2.3–5.6
Telangiectasia	3.8	1.8–5.7
Breast oedema	4.7	2.4–7.0

∗ Locoregional relapse α/β START-A results.

† Locoregional relapse α/β from START-A combined with START-pilot results.

**Table 2:** Summary of the breast cancer hypofractionation trials mentioned in this review

Trial name	Dates	No. patients	Trial arm	Control arm	α/β estimate
START pilot	1986–1998	1410	39 Gy or 42.9 Gy, 13 fractions, 5 weeks	50 Gy, 25 fractions, 5 weeks	3.5–4.7 Gy∗
Ontario	1993–1996	1234	42.5 Gy, 16 fractions, 3 weeks	50 Gy, 25 fractions, 5 weeks	N/A
START-A	1999–2002	2236	39 Gy or 41.6 Gy, 13 fractions, 5 weeks	50 Gy, 25 fractions, 5 weeks	3.5–4.7 Gy∗
START-B	1999–2002	2215	40 Gy, 15 fractions, 3 weeks	50 Gy, 25 fractions, 5 weeks	N/A
FAST	2004–2007	915	28.5 Gy or 30 Gy, 5 fractions, 5 weeks	50 Gy, 25 fractions, 5 weeks	2.3–2.6 Gy∗
RAPID	2006–2011	2135	38.5 Gy, 10 fractions, 5 days (partial breast)	42.5 Gy, 16 fractions or 50 Gy, 25 fractions daily (whole breast)	N/A
FAST Forward	2011–present	4000	26 Gy or 27 Gy, 5 fractions, 5 days	40 Gy, 15 fractions, 3 weeks	Follow-up

∗ Range of α/β values calculated depending on end point used, see Table 1.

**Table 3:** Summary of the prostate cancer hypofractionation trials mentioned in this review

Trial name	Dates	No. patients	Trial arm	Control arm	α/β estimate
“Australian trial”	1996–2003	217	55 Gy, 20 fractions, 4 weeks	64 Gy, 32 fractions, 6.5 weeks	N/A
CHHiP	2002–2006	3216	60 Gy, 20 fractions or 57 Gy, 19 fractions	74 Gy, 37 fractions, 7.5 weeks	Follow-up
PROFIT	2005–2012	1204	60 Gy, 20 fractions, 4 weeks	78 Gy, 39 fractions, 8 weeks	N/A
HYPRO	2006–2011	800	64.6 Gy, 19 fractions, 7 weeks	78 Gy, 39 fractions, 8 weeks	N/A
HYPO-RT-PC	2005–present	592	42.7 Gy, 7 fractions, 3 weeks	78 Gy, 39 fractions, 8 weeks	N/A
PACE	2012–present	1700	36.25 Gy, 5 fractions or 38 Gy, 4 fractions with CyberKnife	78 Gy, 39 fractions, 8 weeks	Recruitment
