# Corrigendum to “Treatment of Breast and Prostate Cancer by Hypofractionated Radiotherapy: Potential Risks and Benefits” [Clin Oncol 27 (7) (2015) 420–426]

**DOI:** 10.1016/j.clon.2015.11.009

**Published:** 2016-02

**Authors:** K.J. Ray, N.R. Sibson, A.E. Kiltie

**Affiliations:** Cancer Research UK and Medical Research Council Oxford Institute for Radiation Oncology, Department of Oncology, University of Oxford, Oxford, UK

The Authors regret that in the above manuscript an acknowledgement to Dr Navita Somaiah was omitted in error. The Authors would like it noted that the following acknowledgement should have been included in the paper:

This is a modified version of Kevin Ray's Radiobiology MSc essay which was based on the topic of Dr. Navita Somaiah's lecture. We thank Dr. Somaiah for very helpful comments on the essay and discussion.

